# Notch2 and Notch3 suppress the proliferation and mediate invasion of trophoblast cell lines

**DOI:** 10.1242/bio.025767

**Published:** 2017-06-12

**Authors:** Wei-Xiu Zhao, Zhen-Ming Wu, Wei Liu, Jian-Hua Lin

**Affiliations:** 1 Department of Obstetrics and Gynecology, South Campus, Ren Ji Hospital, School of Medicine, Shanghai Jiaotong University, Shanghai 201112, China; 2 Department of Obstetrics and Gynecology, Ren Ji Hospital, School of Medicine, Shanghai Jiaotong University, Shanghai 200127, China

**Keywords:** Notch2, Notch3, Trophoblast cell lines, Proliferation, Invasion

## Abstract

Notch signaling pathways play important roles in cell fate and many diseases, including preeclampsia, the dysregulation of which may be the main cause of maternal mortality. This study aimed to investigate the roles of Notch2 and Notch3 in proliferation and invasion in trophoblast cell lines (BeWo and JAR). Small hairpin RNAs targeting Notch2/Notch3 and Notch2/Notch3-overexpression vectors were designed, constructed and transfected into BeWo and JAR cells. Quantitative real-time polymerase chain reaction (qRT-PCR) and western blotting were then used to detect Notch2 and Notch3 mRNA and protein levels, and confirm the efficiency of silence and overexpression. Flow cytometry assays were conducted to evaluate the cell cycle of the two cell lines, and transwell assays were used to detect migration and invasion. Western blot analysis was also performed to show the alteration of the cell lines' physiological activities at protein level.

When Notch2 was downregulated in BeWo cells, proliferation was dramatically promoted, while migration and invasion were significantly inhibited. When Notch2 was upregulated in JAR cells, proliferation was inhibited, but migration and invasion were promoted. After overexpression of Notch3 in BeWo cells, proliferation was downregulated, but migration and invasion were both upregulated. By contrast, the silencing of Notch3 expression in JAR cells significantly enhanced proliferation, but suppressed migration and invasion. These data indicated that Notch2 and Notch3 mediate the invasion and migration of BeWo and JAR cells, and may play a potential role in early onset severe preeclampsia.

## Abstract (precis)

**Summary:** This study reveals some of the potential roles of Notch2 and Notch3 in migration and invasion in trophoblast cells. The results may help to increase our understanding of the pathology of preeclampsia.

## INTRODUCTION

Preeclampsia is a gestation-specific disorder with a high incidence of mother and infant mortality that affects 3.0-5.0% of pregnant women worldwide (Mol et al., 2016). However, effective strategies for the prevention and treatment of the disorder remain lacking (Iriyama et al., 2015). Although the pathogenesis of preeclampsia remains unclear, failures in vascular changes of the placenta bed appear to play a role. Whether the vascular cell differentiation affects the process of placental angiogenesis is not completely clear. The differentiation of cells in human placenta appears to result from close interaction between villous trophoblasts, stroma and vascular endothelium (Kingdom et al., 2000). Trophoblasts are primary cells for implantation and maintenance of pregnancy, and play a crucial role in the formation of placenta and nourishment of the embryo. Basic research has focused on the molecular mechanisms by which trophoblast invasion is controlled under physiological and pathological conditions (Knofler, 2010). Here, we attempted to identify new pathogenic factors linking placental pathology to the development of preeclampsia.

Notch signaling is involved in determining cell fate, cell differentiation and many other biological processes. This pathway plays a vital role in placental development, but the specific mechanisms that regulate Notch signaling pathway remain unknown in human placenta (Zhao and Lin, 2012) The Notch pathway contains four Notch receptors (Notch1-4) and represents a key regulator in the control of trophoblast proliferation and apoptosis (Jaiswal et al., 2015). Four genes, *Notch1-4*, are expressed in a variety of tissues in a relative complementary manner during development. Specification of trophoblast subtypes appears to not be disturbed, and the expression of downstream genes of Notch2 signaling was not altered in *Notch2* mutant mouse placentas (Hamada et al., 2007). However, there is evidence that Notch2 and Notch3 are expressed mainly in cytotrophoblast cells (Cormier et al., 2004). It is hypothesized that abnormal expression of Notch2 and Notch3, and the resulting effects on trophoblast cells, might lead to the early onset of severe preeclampsia and other obstetric disorders (Fragkiadaki et al., 2015).

The results of a previous study showed an obvious upregulation of Notch3 expression, and a downregulation of Notch2 expression, in placentas from patients with early onset severe preeclampsia compared with those in normal placentas (Zhao et al., 2014). Notch2 and Notch3 inhibit both cell proliferation and cell apoptosis in BeWo and JAR cells (Zhao et al., 2015). However, the mechanisms by which Notch2 and Notch3 are related to preeclampsia remain unknown. No studies have addressed the impact of Notch2 and Notch3 on trophoblast cell migration and invasion. The functional regulation of human trophoblast cells by Notch2 and Notch3 also remains unclear. In this study, we investigated the effects of Notch2 and Notch3 on cell migration and invasiveness in trophoblast BeWo and JAR cell lines by knocking down Notch2 or Notch3 with short hairpin (sh)RNA and upregulating Notch2 or Notch3 gene expression. The study was conducted to explore the potential effects of Notch2 and Notch3 on migration and invasion in trophoblast cells.

## RESULTS

### Expression levels of Notch2 and Notch3 in trophoblast cell lines

**Fig. 1.**
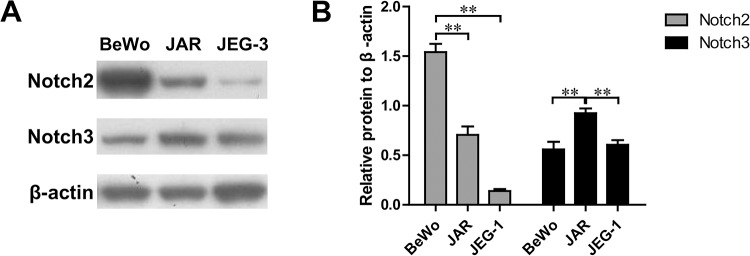
**Notch2 and Notch3 expression in trophoblast cell lines.** (A) Western blotting revealed differences in endogenous Notch2 and Notch3 expression in three human trophoblast cell lines: BeWo, JAR, and JEG-3. β-actin was used as a loading control. (B) Protein levels of Notch2 and Notch3 were quantified by normalizing the intensity for each band with that of β-actin. Data are mean±s.d. **P<0.01.

To study the relationships among Notch2, Notch3 and trophoblast cells, we first examined the levels of Notch2 and Notch3 expression in three trophoblast cell lines: BeWo, JAR and JEG-3. As shown in Fig. 1A,B, both Notch2 and Notch3 could be detected in all three trophoblast cell lines; BeWo cells had the highest Notch2 expression and lowest Notch3 expression, and JAR cells had the highest Notch3 expression, compared to the other two cell lines. The expression of Notch2 is decreased, and that of Notch3 is generally increased, in the placental tissues from patients with preeclampsia compared to those with normal placental tissues, according to previous experiments (Zhao et al., 2014). To determine the associations among Notch2, Notch3 and early onset severe preeclampsia, immunohistochemical staining was performed on the placental tissues of patients with early onset severe preeclampsia (Zhao et al., 2014). These findings suggested that the expression of Notch2 and Notch3 might be closely related to trophoblast cell function and might be an important factor in preeclampsia development. Based on these results, we chose the BeWo and JAR cell lines for use in subsequent studies.

### Efficiency of Notch2 and Notch3 gene knockdown or overexpression

**Fig. 2.**
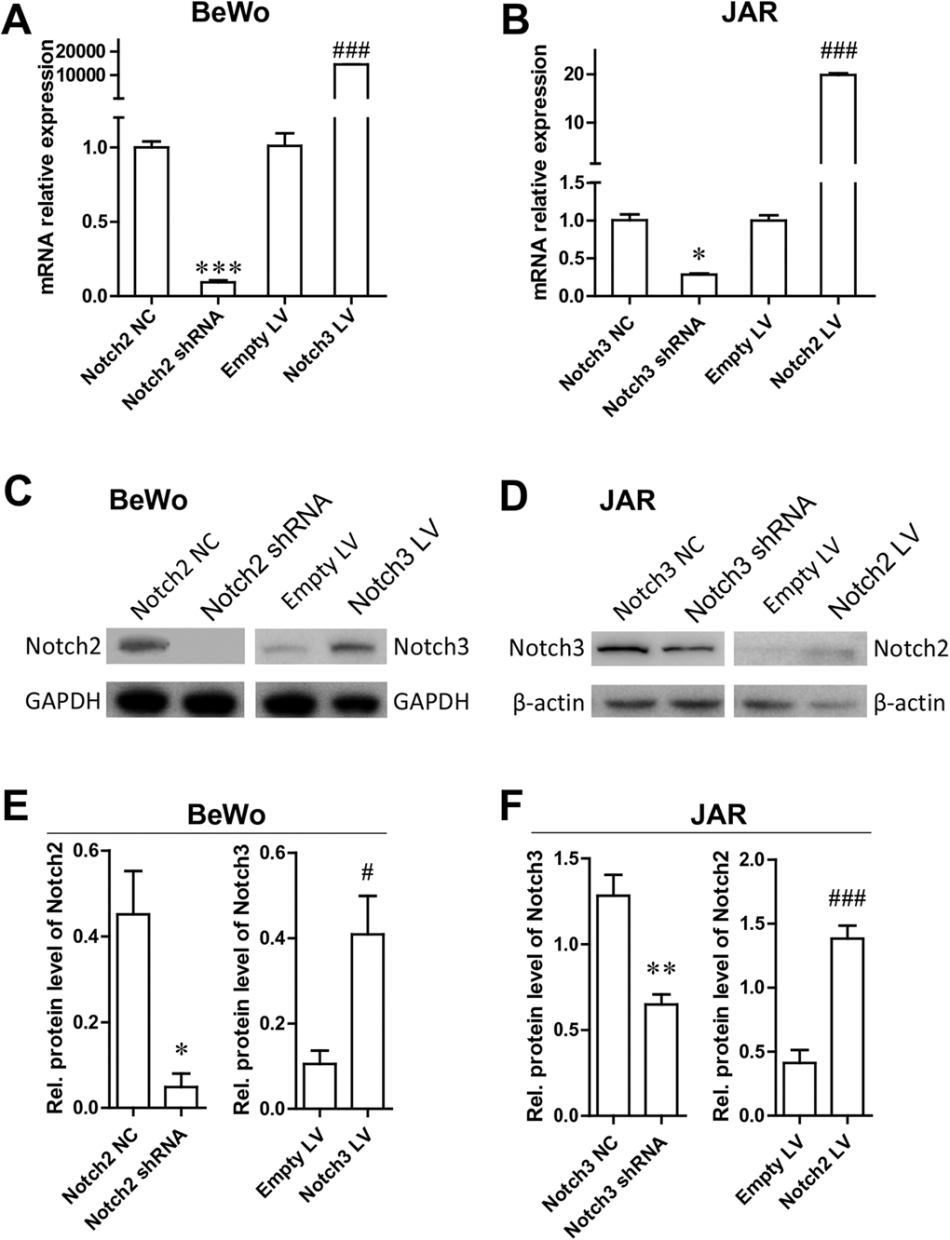
**Notch2 and Notch3 shRNA silencing and expression efficiencies.** (A) qRT-PCR analysis of Notch2 and Notch3 mRNA in BeWo cells with Notch2 knockdown or Notch3 overexpression. (B) qRT-PCR analysis of Notch2 and Notch 3 mRNA in JAR cells with Notch3 knockdown or Notch2 overexpression. (C) Western blot analysis of Notch2 and Notch3 protein in BeWo cells with Notch2 knockdown or Notch3 overexpression. (D) Western blot analysis of Notch2 and Notch3 protein in JAR cells with Notch3 knockdown or Notch2 overexpression. (E,F) Densitometric analysis of Notch2 and Notch3 protein levels in C and D. *P<0.05, **P<0.01, ***P<0.001, compared with the negative control (NC); ^#^*P*<0.05, ^###^*P*<0.001, compared with Empty LV.

Strong Notch2 expression was found in BeWo cells, and high Notch3 expression in JAR cells, so we used shRNA and overexpression vectors to manipulate the expression of Notch2 and Notch3. To evaluate the roles of Notch2 and Notch3 silence and overexpression in preeclampsia subtypes, BeWo and JAR cells were transfected with shRNA or overexpression vectors. Quantitative real-time polymerase chain reaction (qRT-PCR) analysis (Fig. 2A,B) showed that Notch2 and Notch3 shRNA and overexpression vector significantly changed the expression of Notch2 and Notch3 mRNAs in selected cell lines compared to that in the negative control. Western blots confirmed Notch2 knockdown and Notch3 overexpression in BeWo cells after transfection with shRNA and overexpression vector (Fig. 2C,E). The results were in conformity with qRT-PCR results in the BeWo cell line. In JAR cells, Notch2 was upregulated, and Notch3 was downregulated, by the specific shRNA targeting Notch2 or Notch3 overexpression vectors, respectively (Fig. 2D,F).

### Notch2 and Notch3 affect cell proliferation and induce cell cycle arrest

**Fig. 3.**
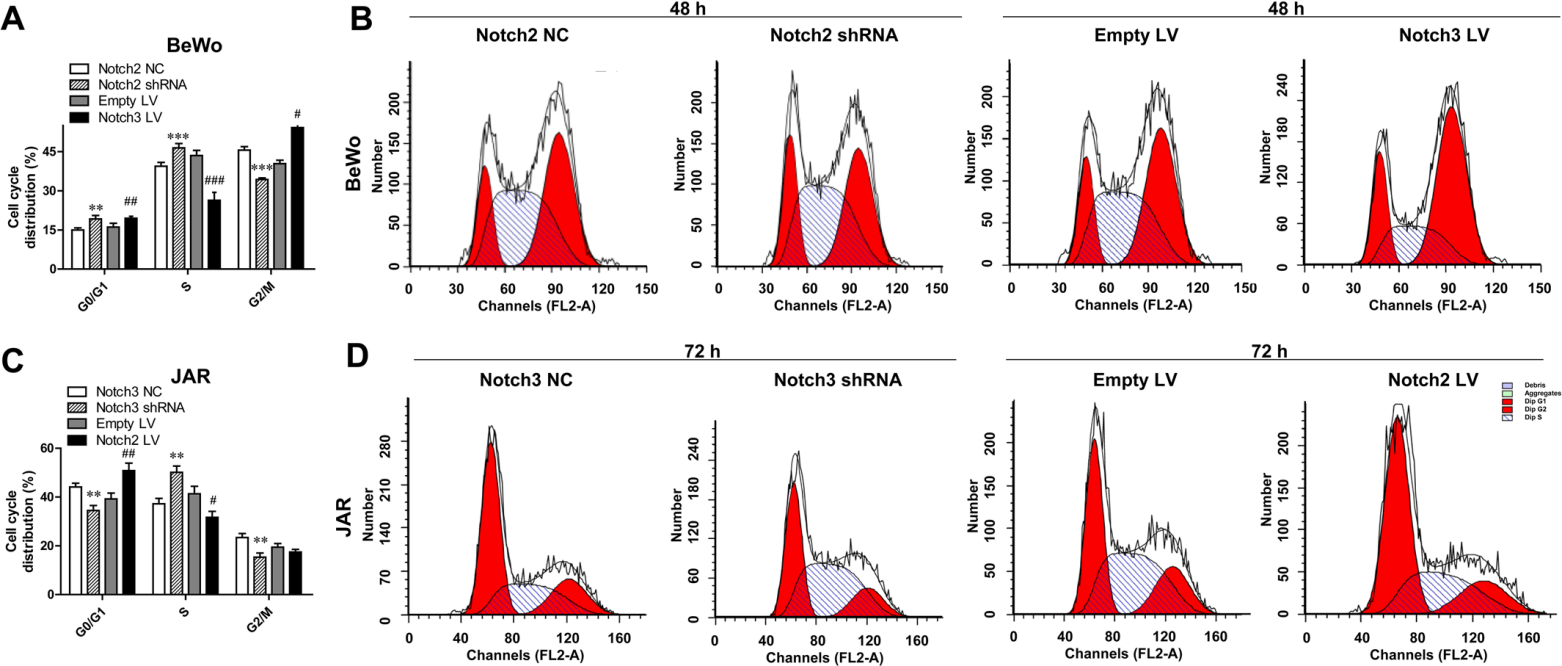
**Altered expression of Notch2 and Notch3 affects the cell cycle of trophoblast cells.** (A,B) Notch2 knockdown arrested BeWo cells in S phase; Notch3 overexpression resulted in the decrease of BeWo cells at S phase, and increase of the cells at G0/G1 and G2/M phases. (C,D) Notch2 overexpression delayed JAR cells in G0/G1 phase. Notch3 knockdown resulted in the decrease of JAR cells in G0/G1 and G2/M phases, and significant cell arrest in S phase. *P<0.05, **P<0.01, ***P<0.001, compared with NC; ^#^*P*<0.05, ^##^*P*<0.01, ^###^*P*<0.001, compared with Empty LV.

Our previously reported results indicated that the expression of Notch2 and Notch3 is associated with the proliferation of BeWo and JAR cells (Zhao et al., 2015). Specifically, Notch2 knockdown significantly promoted BeWo cell proliferation, while Notch3 overexpression decreased the growth rate of BeWo cells, compared with that of the negative control. Meanwhile, Notch2 overexpression inhibited, whereas Notch3 knockdown accelerated, JAR cell proliferation. To explore whether Notch2 and Notch3 expression has an influence on the cell cycle, we investigated the effects of Notch2 silence or Notch3 overexpression on BeWo cells, and Notch3 silence or Notch2 overexpression, on JAR cells. Fluorescence-activated cell sorting (FACS) results indicated that Notch2 knockdown significantly increased the percentage of BeWo cells in the S phase (*P*<0.001), and that Notch3 overexpression decreased the percentage of BeWo cells in the S phase and induced the cells to stop at G0/G1 and G2/M phases (*P*<0.001) (Fig. 3A,B), compared to the negative control shRNA or empty vector (empty LV). The percentage of JAR cells with reduced endogenous Notch3 significantly increased in the S phase (*P*<0.01) and significantly decreased in G0/G1 and G2/M phases (*P*<0.01) (Fig. 3C,D). The percentage of JAR cells with Notch2 overexpression increased in the G0/G1 phase and decreased in the S phase (Fig. 3C,D). Notch3 shRNA in JAR cells could thus promote cells to continue their cycle from the G0/G1 to the S phase. These data demonstrated that upregulation of Notch2 and Notch3 might inhibit BeWo and JAR cell growth.

### Notch2 and Notch3 are necessary for cell migration and invasion of trophoblast cells

**Fig. 4.**
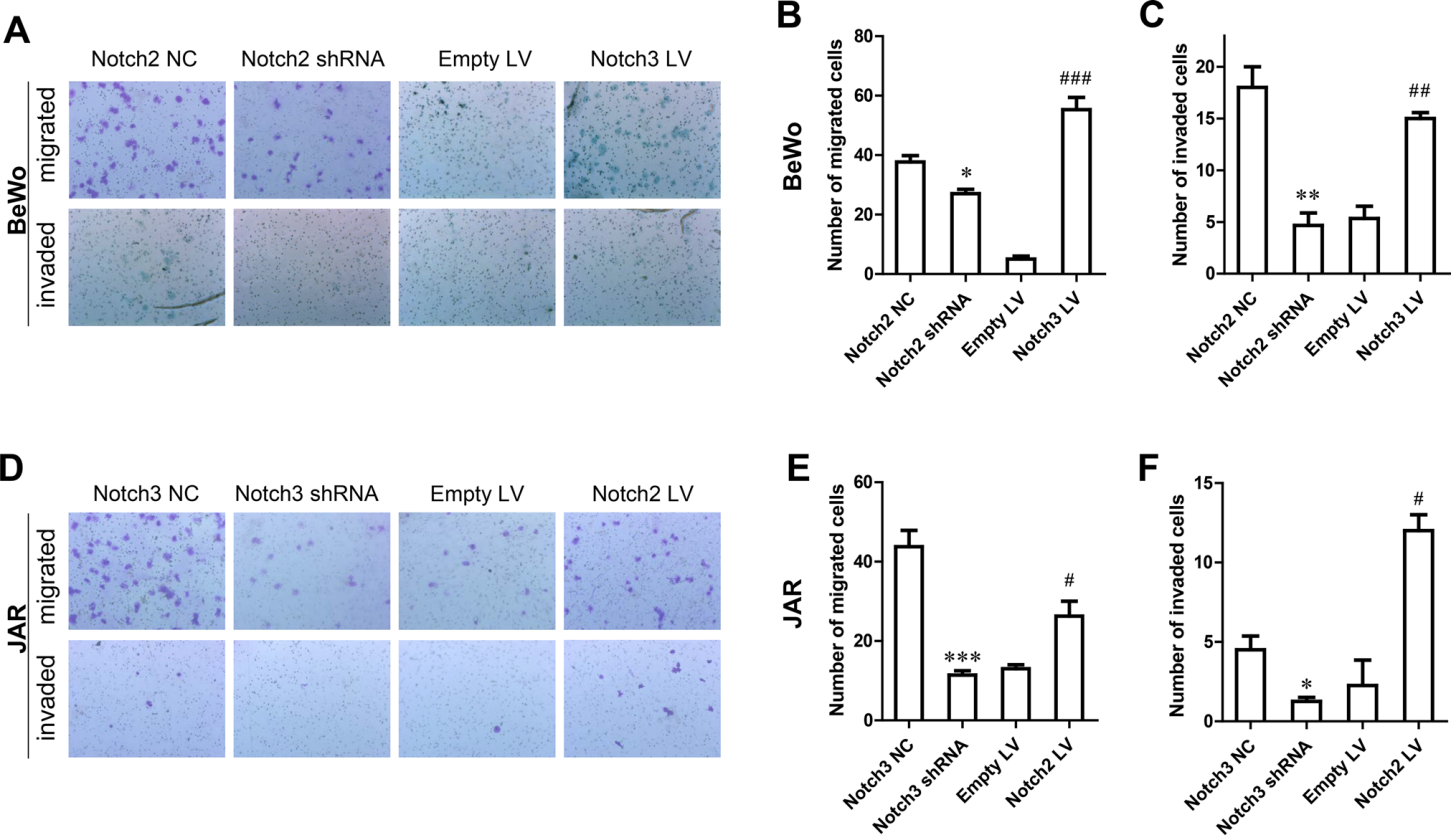
**Effects of Notch2 and Notch3 on trophoblast cell migration and invasion.** (A) Migration and invasion assay of BeWo cells transfected with Notch2 shRNA (magnification 100×). (B) The number of migrated BeWo cells in the migration and invasion assay. (C) The number of invaded BeWo cells in the migration and invasion assay. (D) Migration and invasion assay of JAR cells transfected with Notch3 shRNA (magnification 100×). (E) The number of migrated JAR cells in the migration and invasion assay. (F) The number of invaded JAR cells in the migration and invasion assay. Cell numbers represent mean±s.d. values per field (from at least five fields) from independent experiments. *P<0.05, **P<0.01, ***P<0.001, compared with NC; #*P*<0.05, ^##^*P*<0.01, ^###^*P*<0.001, compared with Empty LV.

To further determine whether Notch2 and Notch3 affect trophoblast cell migration and invasion, transwell migration and invasion assays were performed. In BeWo cells, knockdown of Notch2 resulted in effective inhibition of migration and invasion compared with the negative control, while overexpression of Notch3 significantly enhanced migration and invasion compared with the empty LV (Fig. 4A,B,C). Consistently, Notch3 knockdown inhibited, but Notch2 overexpression promoted, JAR cell migration and invasion (Fig. 4D,E,F). However, the significant phenotypical differences between cells with negative control (NC) shRNA and empty LV were possibly due to excessive viral load upon ectopic infection. These results demonstrated that Notch2 and Notch3 might both actively participate in migration and invasion in trophoblast cells.

### Notch2 and Notch3 effectively regulate downstream targets *in vitro*

**Fig. 5.**
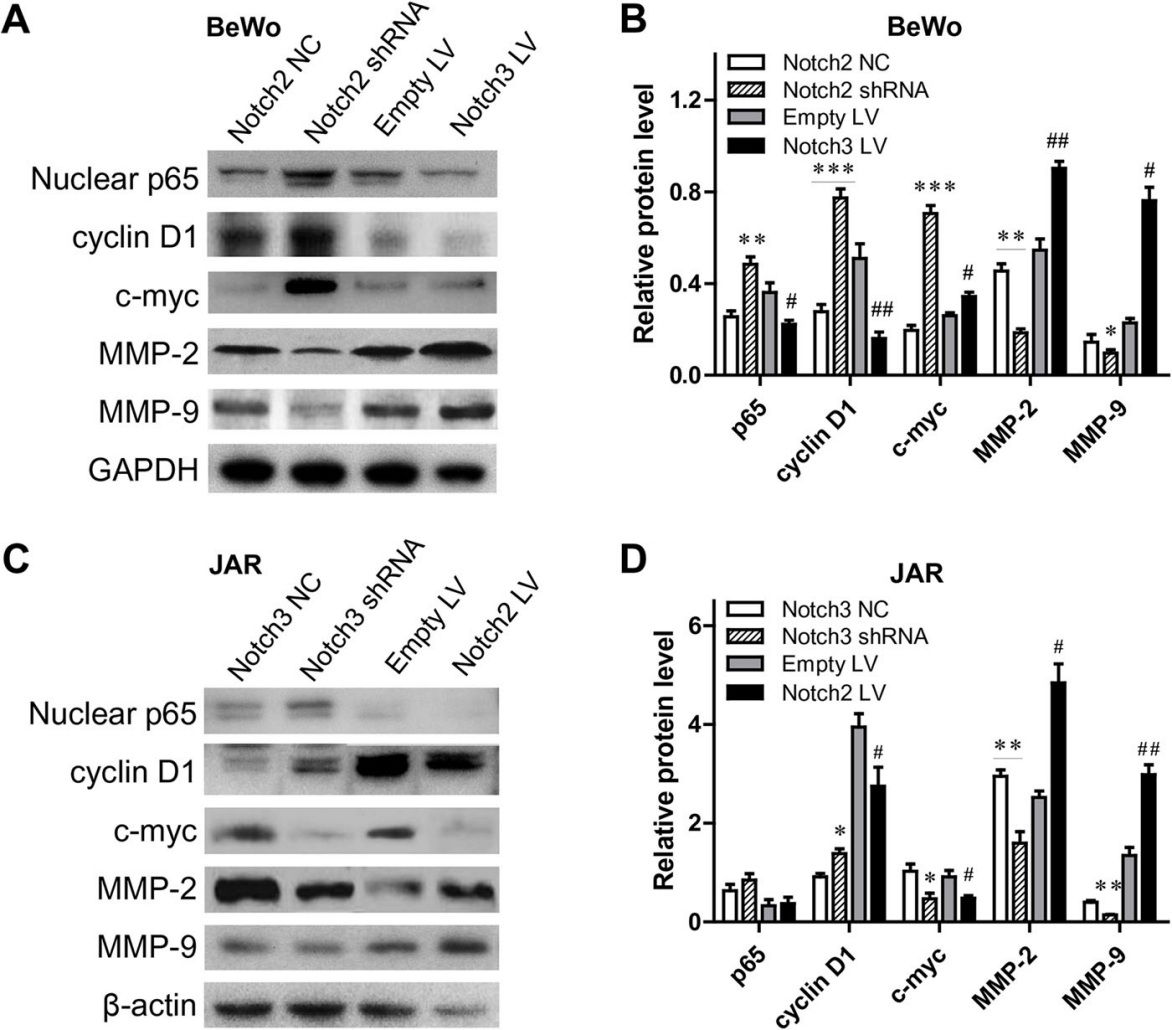
**Effects of Notch2 and Notch3 on downstream targets in trophoblast cells.** (A) Western blots of nuclear p65, cyclin D1, c-Myc, MMP-2 and MMP-9 protein expression in Notch2-knockdown or Notch3-overexpressed BeWo cells. (B) Levels of nuclear p65, cyclin D1, c-Myc, MMP-2 and MMP-9 protein were quantified by normalizing the intensity of each band with that of GAPDH. (C) Western blots of nuclear p65, cyclin D1, c-Myc, MMP-2 and MMP-9 protein expression in Notch3-knockdown or Notch2-upregulated JAR cells. (D) Levels of nuclear p65, cyclin D1, c-Myc, MMP-2 and MMP-9 protein were quantified by correcting the intensity of each band with that of β-actin. *P<0.05, **P<0.01, ***P<0.001, compared with NC; ^#^*P*<0.05, ^##^*P*<0.01, ^###^*P*<0.001, compared with Empty LV.

We investigated the potential molecular mechanism affected by Notch2 and Notch3 in trophoblast cells by evaluating the expression of NF-κB p65, cyclin D1, c-Myc, matrix metalloproteinase (MMP)-2 and MMP-9 in the trophoblast cells after Notch2 and Notch3 knockdown or overexpression. Western blot analysis showed that nuclear p65, cyclin D1 and c-Myc proteins significantly increased, whereas MMP-2 and MMP-9 decreased, in Notch2-silenced BeWo cells (Fig. 5A,B). When Notch3 was overexpressed in BeWo cells, nuclear p65 and cyclin D1 expression notably decreased, whereas c-Myc, MMP-2 and MMP-9 expression increased. Notch3 knockdown in JAR cells promoted cyclin D1 expression and downregulated c-Myc, MMP-2 and MMP-9 protein levels (Fig. 5C,D). Upregulated Notch2 in JAR cells obviously increased cyclin D1, MMP-2 and MMP-9 protein levels, and decreased the expression of c-Myc (Table 1). The change in cyclin D1 was consistent with the cell cycle results of the BeWo cells and JAR cells. We considered that cyclin D1, c-Myc, MMP-2 and MMP-9 might play prominent roles in mediating cell proliferation and invasion in trophoblast cells with overexpression or knockdown of Notch2 and Notch3.

**Table 1.**
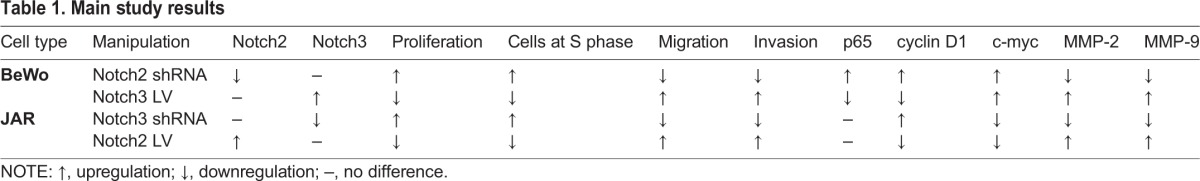
**Main study results**

## DISCUSSION

It is generally accepted that trophoblast invasion is a multistep process. Trophoblast cells experience an epithelial–mesenchymal transition when an embryo attaches to the endometrium, to form multilayered cell columns and then invade the blood vessels and decidual stroma (Hunkapiller et al., 2011; Chen et al., 2012). Subsequently, the endothelial cells are replaced by the fibrinoid endovascular trophoblasts which have invaded the arteries. Finally, spiral artery remodelling is completed. Notch signaling has been identified as a key regulatory pathway controlling trophoblast proliferation, motility and differentiation (Haider et al., 2014). It has been proven that early onset severe preeclampsia is related to trophoblastic cell migration and invasion (Yang et al., 2015). Previous research showed that Notch2 expression decreased, and Notch3 was overexpressed, in the placentas of patients with preeclampsia compared with normal placentas (Zhao et al., 2014), which indicate that Notch2 and Notch3 may play important roles in the pathology of preeclampsia. In our previously published research, we demonstrated that Notch2 and Notch3 exert antiproliferative effects on trophoblast cells (Zhao et al., 2015). In the present study, we present more evidence that high levels of Notch2 and Notch3 are able to induce cell cycle arrest at G2/M and G0/G1 phases, subsequently resulting in suppressed proliferation. In other studies, Notch1 gene silencing had been shown to reduce the invasiveness of trophoblasts (Yu et al., 2014), but there is still no research about the roles of Notch2 and Notch3 in the migration and invasiveness of trophoblast cells. In this study, we showed that the expression of both Notch2 and Notch3 were positively correlated with migration and invasion in BeWo and JAR cells, proving that Notch2 and Notch3 might be indispensable for the migration and invasion of the trophoblast cells.

Trophoblast invasion is strictly controlled by various proteolytic enzymes, such as MMPs, especially members of the gelatinase family, including MMP-2 and MMP-9. In early gestation, MMP-2 and MMP-9 are highly expressed by trophoblasts, and the measurement of their activities provides an indicator of trophoblast cell invasiveness (Halasz and Szekeres-Bartho, 2013). Our study results showed that MMP-2 and MMP-9 are downregulated after Notch2 knockdown, and upregulated when Notch2 is overexpressed, in BeWo cells and JAR cells. The observed change in MMP-2/9 protein is consistent with the influence of Notch2 on invasiveness. The nuclear transcription factor NF-κB regulates cell behavior in several ways, such as by increasing proliferation, inhibiting apoptosis, and increasing inflammatory and immune responses (Rahardjo et al., 2014). Activated NF-κB also plays an important role in the physiological activities underlying the development of trophoblast cell cancer, such as it is activated nearly 10-fold in women with preeclampsia (Vaughan and Walsh, 2012). The level of nuclear p65 is increased in BeWo and JAR cells with Notch2 and Notch3 knockdown. Activation of the Notch signaling pathway induces genes such as c-Myc, cyclin D1 and MMPs (Haider et al., 2014). High levels of c-Myc mRNA and protein expression in cytotrophoblasts have been detected (Kumar et al., 2013), which modulate cellular processes, including proliferation, growth, apoptosis and differentiation. The Myc oncoprotein regulates tumor metastasis through specific effects on cancer cell invasion and migration (Wolfer and Ramaswamy, 2011). In the human placenta, c-Myc was observed to peak at 4.0-5.0 weeks' gestation and to be highly expressed in the proliferating trophoblast (Kumar et al., 2013). The expression of c-Myc is significantly increased after Notch2 knockdown, and decreased after Notch2 overexpression, in the two cell lines investigated in our study.Therefore, c-Myc might mediate cell proliferation in the Notch2 pathway. Trophoblast and tumor cells have similar invasion mechanisms and share similar biochemical mediators, such as c-Myc and MMP-9 (Kitroser et al., 2012). Although there is a normal progression of trophoblasts into the placental bed, the trophoblasts' failure to invade and migrate into the arteries appears to be a significant feature of preeclampsia pregnancy (Kharfi et al., 2003). Invading trophoblastic cells lead to significant structural changes in uterine blood vessels, which can induce interference in complicated pregnancies. In the present research, we investigated the effects of regulation of Notch2 and Notch3 on the migration and invasiveness of BeWo and JAR cells. Our studies suggested that the migration and invasion of BeWo cells reduced after Notch2 knockdown, and that Notch2 overexpression promoted the migration and invasion of JAR cells.

It has been proven that Notch3 expression is significantly increased in the placenta of patients with preeclampsia (Zhao, et al., 2014), given that Notch3 enhances the migration and invasion of cells in the placenta. Our study results showed that Notch3 overexpression enhanced the migration and invasion of BeWo cells, and Notch3 knockdown reduced the migration and invasiveness of JAR cells. The level of c-Myc, MMP-2 and MMP-9 increased, and cyclin D1 expression decreased, when Notch3 was upregulated. The change in MMP-2 level was consistent with the change in invasiveness upon Notch3 knockdown or overexpression. c-Myc protein levels decreased in JAR cells with Notch3 knockdown, similar to the change in invasiveness, but cyclin D1 expression increased. So, c-Myc may play different roles in Notch2 and Notch3 pathways, which mediate cell migration and invasion in the Notch3 pathway. It might be considered that this inconsistency within the placenta could compensate for the adverse effects caused by Notch2 downregulation. In addition, considering the cells used in this experiment, trophoblast cell lines cannot completely replace primary trophoblast cells, which is also a limitation in this research field. Although immunohistochemistry showed that Notch3 is expressed mainly in placental trophoblast cells, there might be similar results if cytotrophoblast cells in the preliminary experiment were isolated and the Notch3 expression changes in these cells were detected.

Taken together, our study results provide evidence that Notch2 and Notch3 are able to suppress proliferation by inducing cell cycle arrest, and promote the migration and invasion of trophoblast cells, through which they might participate in the pathogenesis of preeclampsia. However, due to limitations in the research, further studies are necessary to unveil the mechanistic roles of these factors in the pathogenesis of early onset severe preeclampsia.

## MATERIALS AND METHODS

### Cell lines and culture

Human choriocarcinoma cell lines, BeWo, JAR and JEG-3 were purchased from the American Type Culture Collection (ATCC, Manassas, VA, USA), and cultured in Dulbecco's Modified Eagle Medium (Life Technologies) containing 10% (vol/vol) fetal bovine serum in an incubator containing 5.0% CO_2_ at 37°C.

### Knockdown or overexpression of Notch2 and Notch3

Short hairpin (sh) RNA and overexpression vectors were designed and synthesized by Shanghai GenePharma (Shanghai, China). The target sites and sequences are listed in Table 2, in which, Notch2-sh7386 (5′-GCAGGTAGCTCAGACCATTCT-3′) and Notch3-sh235 (5′-ATCTCCAGCATTACTACCGAG-3′) were selected to clone into the lentivirus vectors because of their effective knockdown effect, and were subsequently renamed Notch2 shRNA and Notch3 shRNA, respectively. The specificity of the shRNAs had been analyzed using the online tool Nucleotide BLAST (https://blast.ncbi.nlm.nih.gov/Blast.cgi), and the results showed that the shRNAs chosen can specifically target Notch2 or Notch3 in humans. For the overexpression of Notch2 and Notch3, their full-length sequences were cloned into the lentivirus vector, and the empty vector was used as negative control (designated as empty LV). BeWo and JAR cells were divided into four groups as follows: shRNA (transfected with Notch2 shRNA or Notch3 shRNA), mock (transfected with negative control RNA), overexpression (transfected with Notch2 or Notch3-vector), and negative control (transfected with empty vector). Cell transfection methods were the same as described previously (Zhao et al., 2015).

**Table 2.**
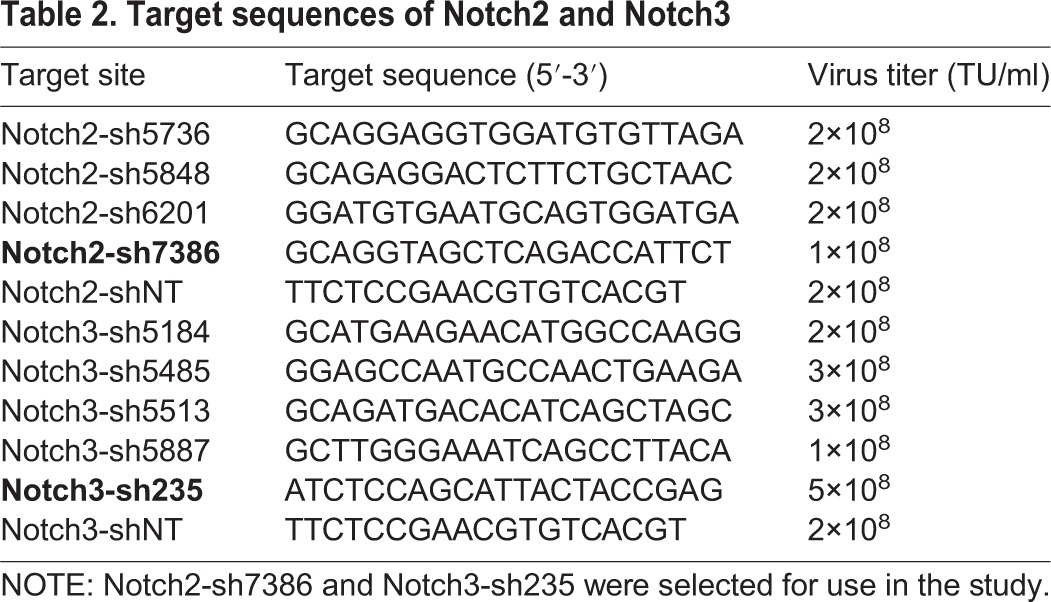
**Target sequences** **of Notch2 and Notch3**

### RNA extraction and qRT-PCR

Infected BeWo and JAR cells were cultured in six-well plates. After 24 h, the culture medium was removed and the cells were washed twice with phosphate buffered saline (PBS). Total RNA was isolated using Trizol reagent (Life Technologies) according to the manufacturer's instructions. Total RNA was purified from cell cultures using the FastQuant RT Kit (TIANGEN Biotech, Beijing, China) and 2.0 μg of the total RNA was converted to cDNA by reverse transcription. Primers used were as follows: Notch 2 forward, 5′-TCAACTGCCAAGCGGATGT-3′ and Notch 2 reverse, 5′-CTTGGCTGCTTCATAGCTCC-3′; Notch 3 forward, 5′-GCTCAACGGCACTGATCCT-3′ and Notch 3 reverse, 5′-AGCCCAGTGTAAGGCTGATT-3′; and GAPDH forward 5′- CGGAGTCAACGGATTTGGTCGTATTGG-3′ and GAPDH reverse, 5′- GCTCCTGGAAGATGGTGATGGGATTTCC-3′. qRT-PCR was performed using the ABI 7500 detection system (Applied Biosystems, Foster City, CA, USA) with SuperRealPreMix Plus (TIANGEN Biotech) to determine the levels of Notch2 and Notch3 mRNA expression. Expression levels were normalized to the mRNA expression of glyceraldehydes 3-phosphate dehydrogenase (GAPDH).

### Western blotting

Western blotting was performed as previously described (Zhao et al., 2014). The blots were probed with the following primary antibodies: anti-Notch2 (Cell Signaling Technologies) at 1:1000 dilution, anti-Notch3 (Santa Cruz Biotechnology) at 1:200, anti-NF-κB p65 (Cell Signaling Technologies) at 1:1000, anti-cyclin D1 (Cell Signaling Technologies) at 1:1000, anti-c-Myc (Abcam,) at 1:5000, anti- MMP-2 (Abcam) at 1:1000, anti-GAPDH (Affinity Biosciences, Cincinnati, OH, USA) at 1:5000, and anti-β-actin (KangChen, Shanghai, China) at 1:5000. Blots were detected using the Western Chemiluminescent HRP Substrate (Merck Millipore, Billerica, MA, USA) and visualized by FluorChem^®^HD2 (ProteinSimple, San Jose, CA, USA).

### Cell cycle analysis

After transfection, the cells were obtained, washed with PBS, and fixed with 70% ice-cold ethanol overnight at 4.0°C. The fixed cells were washed with cold PBS, then incubated with PBS and 500 μl PI/RNase Staining Buffer (BD Biosciences) in the dark for 30 min at 4.0°C. The contents of the labeled cells were determined using a flow cytometer (BD FACScan, BD Biosciences) with CellQuest acquisition and analysis programs.

### *In vitro* cell migration and invasion assays

A cell migration assay was conducted using a transwell chamber (24-well, 8.0-μm pore membranes; BD Biosciences). For the invasion assay, 5.0 mg/ml BD Matrigel basement membrane matrix (BD Biosciences) was added to the upper membrane and allowed to gel for 40 min at 37°C. The transwell migration assay was also prepared by incubating the trophoblast cells in 50 μl serum-free medium for 30 min at 37°C, after which 600-800 μl medium containing 10% FBS was placed in the lower chamber. Cells in the serum-free medium were introduced into the upper chamber of quadruplicate wells at a density of 2.0×10^5^ cells/ml (for both migration and invasion assays). After being incubated at 37°C for 24 h, the noninvasive cells from the upper chamber were completely removed using cotton swabs. The invasive cells attached to the lower membrane surface were fixed with paraformaldehyde and stained with Crystal Violet for a minimum of 10 min. The number of invasive cells was counted with an Olympus IX71 microscope (Olympus Corporation, Tokyo, Japan). Each experiment was conducted in triplicate.

### Statistical analyses

All experiments were independently repeated at least three times, and the statistics were calculated based on triplicate data. Values are presented as mean±standard deviation (s.d.) and were analyzed by an independent samples *t*-test. The statistical analyses were performed using SPSS 17.0 (SPSS Inc., Chicago, IL, USA).) A statistically significant difference was defined as *P*<0.05.
