# Expression of Concern: Notch2 and Notch3 suppress the proliferation and mediate invasion of trophoblast cell lines

**DOI:** 10.1242/bio.060225

**Published:** 2023-12-05

**Authors:** Wei-Xiu Zhao, Zhen-Ming Wu, Wei Liu, Jian-Hua Lin

There are potential issues in Figs 2 and 5 in *Biol. Open* (2017) **6**, 1123-1129 (doi:10.1242/bio.025767).

It appears that there may be duplication of the GAPDH blots in Figs 2C and 5A and the β-actin blots in Figs 2D and Fig 5C.

We have made multiple attempts to contact all authors to request original data for the affected figures but have not received a response. Therefore, the journal is publishing this note to alert readers to our concerns.

